# Impact of Omega-3 Supplementation on High Sensitive C-Reactive Protein Level and 30-Day Major Adverse Cardiac Events After the Implementation of Coronary Stent in Patients with Chronic Kidney Disease: A Randomized Clinical Study

**DOI:** 10.15171/apb.2018.055

**Published:** 2018-08-29

**Authors:** Farzaneh Foroughinia, Bahram Movahed Nouri, Javad Kojuri, Mohammad Ali Ostovan

**Affiliations:** ^1^Clinical Neurology Research Center, Shiraz University of Medical Sciences, Shiraz, Iran.; ^2^Clinical Pharmacy Department, Shiraz University of Medical Sciences, Shiraz, Iran.; ^3^Student Research Committee, Shiraz University of Medical Sciences, Shiraz, Iran.; ^4^Cardiology Department, Shiraz University of Medical Sciences, Shiraz, Iran.

**Keywords:** Chronic kidney disease, High sensitive C-reactive protein, Major adverse cardiac events, Omega-3 supplement, Percutaneous coronary intervention

## Abstract

***Purpose:*** Studies have revealed that patients with chronic kidney disease (CKD) are more susceptible to adverse effects of percutaneous coronary intervention (PCI). In addition, the role of elevated high sensitive C-reactive protein (hs-CRP) in the prediction of adverse cardiac outcomes after coronary stent implantation has already been shown. Therefore, in this study, we aimed to evaluate the effect of omega-3 supplementation on hs-CRP and 30-day major adverse cardiac events (MACE) in patients with CKD undergoing elective PCI.

***Methods:*** In this randomized trial, 80 CKD patients who were candidates for elective PCI, were randomly assigned to two groups; the first group received a single dose of omega-3 (2500 mg, 12 h before PCI) as well as the standard drug regimen of PCI and the second group received placebo plus the standard therapy (325 mg loading dose of aspirin, 600 mg loading dose of clopidogrel, and weight-adjusted intravenous heparin). Hs-CRP levels were measured at baseline and 24 h after the intervention as a primary endpoint. The secondary endpoint was the incidence of MACE over a 30-day period after PCI.

***Results:*** Omega-3 did not significantly decrease post-PCI serum level of hs-CRP; however, the overall 30-day MACE was significantly lower in the omega-3 group compared to the control group (p=0.05).

***Conclusion:*** Our results revealed the positive effect of the omega-3 supplement on decreasing 30-day MACE; hence, omega-3 may be considered as an effective adjunctive therapy to the standard drug regimen used before PCI. The evaluation of the effect of omega-3 on long-term MACE is recommended for future studies.

## Introduction

Today, coronary artery diseases are the main cause of death in patients with chronic kidney disease (CKD), and it is estimated that the mortality rate of these patients can be as high as 59% in the first year following a myocardial infarction. CKD patients are not only more likely suffer from cardiac diseases but also are at higher risk of experiencing severe adverse cardiac events.^1^ Moreover, recent studies indicate that CKD patients undergoing percutaneous coronary intervention (PCI) are more susceptible to various adverse effects such as intervention failure, longer length of stay in hospitals, and higher mortality after PCI.^2^

The prognostic value of cardiac necrosis markers, troponins and creatine kinase-MB, in the prediction of complications of PCI, has been described extensively in the literature. Therefore; it is recommended to use these values as a marker of baseline risk, atherosclerosis burden, and procedural complications in the setting of coronary re-vascularization.^3^ On the other hand; several studies have indicated the key role of inflammation in the pathogenesis of atherosclerosis and its progression. Coronary angioplasty and coronary stent implantation have been shown to induce an inflammatory response automatically.^4^ Inflammatory markers such as high sensitive C-reactive protein (hs-CRP), a non-specific but sensitive marker of inflammation, have been reported to be able to predict the cardiovascular risk in a variety of clinical settings such as PCI. Several studies have demonstrated the potential role of elevated hs-CRP in the prediction of both early and late adverse cardiac outcomes after coronary stent implantation.^4,5^ Also, serum hs-CRP level has been shown to be a strong predictor of both restenosis and major adverse cardiac events (MACE) in patients with CKD who have undergone PCI.^6^ As a result; this marker may be of value in the evaluation of therapies investigated to decrease MACE after PCI.

Given the beneficial effects of omega-3 polyunsaturated fatty acids (PUFAs), such as anti-inflammatory, antiplatelet, and its alleviating effect on aspirin responsiveness in drug-resistant patients,^7^ this study aimed to evaluate the effect of pre-treatment with a single dose of omega-3 on post-PCI hs-CRP level and 30-day MACE in CKD patients. To the best of our knowledge, this is the first trial on the impact of omega-3 in reducing post-PCI hs-CRP and cardiac complications in CKD patients.

## Materials and Methods

### Patients and study design 

This study is a pilot, prospective, double-blind, and randomized clinical trial undertaken from January 2015 to January 2016 in the cardiac catheterization laboratory of two tertiary cardiac care centers. Patients, main investigator, and statistician were blind to the allocation.

Inclusion criteria were an age range of 15–80 years, non ST-elevated acute coronary syndrome, candidates for elective PCI, and an estimated glomeration filtration rate (eGFR) between 30-60 mL/min/1.73 m2. Exclusion criteria were a history of cardiac bypass surgery in the last 3 months, history of significant gastrointestinal or genitourinary bleeding in the last 6 weeks, administration of bivalirudin and glycoprotein IIb/IIIa inhibitors during PCI, recent supplementation of omega-3 in the last one month, signs of active bleeding, platelet count < 70 * 10^9^/L, allergy to aspirin, clopidogrel or omega-3, and unsuccessful PCI.

CKD was defined as an eGFR under 60 mL/min/1.73 m2. To identify CKD cases, patientsˈ medical records were evaluated and eGFRs were estimated in the ones with previous history of CKD. eGFR was calculated using Cockcroft-Gault equation: CrCl (ml/min) = {([140-age] × weight [kg])/ 72 × serum creatinine (ml/min)} (×0.85 for women) (2). Pre-procedural serum creatinine, obtained shortly before the PCI, was used for calculating eGFR.

### Medical treatment 

Eligible patients were randomly assigned to two groups using simple randomization method. All patients received single or multiple drug-eluting stents (DES) for various lesions. The control group received placebo (edible paraffin) plus pre-PCI standard therapy while the omega-3 group received standard therapy together with omega-3 supplement. In accordance with the institutional protocols, all patients were pretreated with oral aspirin (325 mg loading dose and then 80 mg/d for the rest of their life) and clopidogrel (600 mg loading dose and then 75mg/d for at least one year after PCI). In addition, patients received weight-adjusted intravenous heparin with a target activated clotting time of 250–350 seconds before the intervention in both groups.

Omega-3 pearls (SUPER NATURAL®; NUTRALAB, Canada) containing 1250 mg fish oil [600 mg eicosapentaenoic acid (EPA) plus 300 mg docosahexaenoic acid (DHA)] were used. Two pearls containing 2500 mg fish oil (1200 mg EPA plus 600 mg DHA) was administered 12 h before PCI for all patients in the omega-3 group. In our previous study, it was shown that pretreatment with a loading dose of omega-3 (1200 mg EPA plus 400 mg DHA) could significantly decrease the CRP levels elevated early after PCI.^8^

### Study endpoints

The primary endpoint was changes in post-PCI hs-CRP between the groups. To measure hs-CRP, blood samples were collected at baseline and 24 h after PCI as a primary endpoint. The secondary endpoint was the incidence of MACE over a period of 30 days after PCI. MACE is defined as myocardial infarction, revascularization treatment, and all-cause death. The follow-up study was carried out by telephone interviews to gather information from the patients.

### Statistical analysis

Statistical analysis was performed by a statistician who was blind to the study, using Statistical Package for Social Sciences software (SPSS version 21.0). Categorical data were expressed as percentages and Pearson chi-square was used to measure the differences between the groups when required assumptions were met; otherwise, the Fisher’s exact test was used. Continuous variables were presented as median (interquartile range) and were analyzed by Mann–Whitney U-test. A p-value of less than 0.05 was considered as statistically significant.

## Results and Discussion

**Figure 1:**
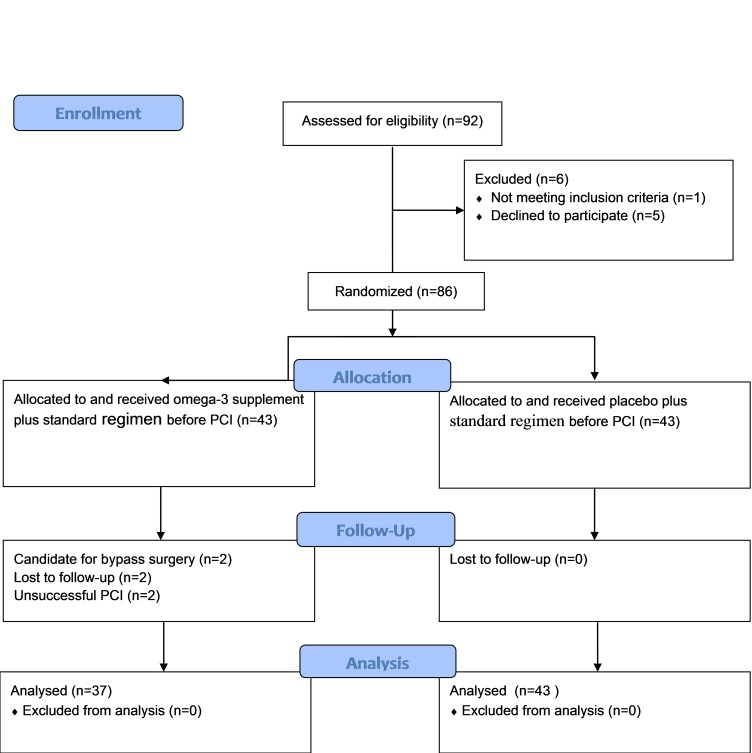
Flow diagram of the trial. Legend: Randomized, double-blind, parallel-design, two-armed study (registered trial IRCT2014122720441N1). The 86 eligible subjects were randomly assigned to receive either omega-3 plus standard therapy (n = 43) or placebo plus standard therapy (n = 43). The measurements for hs-CRP level were performed at 24 h with 80 participants (n = 37 omega-3 group and n = 43 control group) while 6 subjects withdrew. At 30-day, 80 participants (n = 37 omega-3 group and n = 43 control group) were followed for major adverse cardiac events.

Overall, 92 CKD patients admitted to two cardiac hospitals were involved in the study. Six patients were excluded before randomization: 5 patients refused to participate and 1 had active bleeding before surgery. Among 43 patients in the omega-3 group, 6 participants were excluded after randomization: 2 were the candidates for cardiac by-pass surgery, 2 did not attend the follow-up, and 2 had unsuccessful PCI (Figure 1).

At the end, 80 patients completed the trial (37 and 43 patients were assigned to the omega-3 and control group, respectively). As shown in Table 1, the two groups had identical basic clinical characteristics including age, sex, body weight, history of the disease, clinical conditions, pre-hospital and in-hospital medication (all *p* > 0.05) except for the eGFR that was significantly lower in the omega-3 group (*p* = .05). All patients were successfully treated by implantation of drug-eluting stents.

Procedural features related to the type and numbers of target vessels in the study groups are reported in Table 2. No statistically significant differences were found between the two groups except for the distribution of left anterior descending (LAD) vessel, which was significantly higher in the omega-3 arm.

As reported in Table 3, no significant difference was observed between the baseline and 24-h hs-CRP levels among the study groups. In addition, there was no significant difference between the baseline and 24-h hs-CRP measurements (*P*=0.432) when comparing the groups; however, a less upward trend was shown in the omega-3 group.

Regarding the 30-day MACE, 10 (27.9%) patients in the control group and 4 (10.8%) in the omega-3 group experienced major cardiac events such as myocardial infarction and need for revascularization procedures. No all-cause death occurred in both groups. Consequently, there was significant difference between the two groups with regards to the total MACE (*p*=0.05) (Table 4).

Following patients for serious adverse effects of the therapy, no major bleeding (e.g. gastrointestinal bleeding, intracranial hemorrhage) was observed. Four (10.8%) patients in the omega-3 group developed hematoma at the femoral site, which was managed simply by applying pressure to the site. No patients in the control arm experienced this complication (*p* = 0.04).

As discussed in detail, this randomized pilot trial was performed to evaluate the effect of pre-treatment with a loading dose of the omega-3 supplement on the inflammatory marker, hs-CRP, and 30-day MACE in CKD patients undergoing PCI. The review of the current literature showed scanty data in this subject.

The main findings of this study were as follow: A) Less upward trend in hs-CRP level after 24 h was seen in the omega-3 group compared to the controls; however, this difference was not significant; B) Significant variations were detected between the study groups with regards to 30-day MACE, with omega-3 group showing fewer adverse events.

**Table 1:** Demographic information and clinical features in the Omega-3 and control groups

**Variable**	**Control (n=43)**	**Omega 3 (n=37)**	**P-value**
Male sex, N (%)	24(55.8%)	24 (64.9%)	0.41
Age, yrs, mean±SD	65.39±9.38	66.72±9.75	0.53
Weight, kg, mean±SD	66.00±8.15	64.96±10.43	0.62
Diabetes mellitus, N (%)	16(37.2)	12(32.4)	0.65
Hypertension, N (%)	34(79.1)	29(78.4)	0.94
Dyslipidemia, N(%)	25(58.1)	24(64.9)	0.53
Smoker, N (%)	15(34.9)	13(35.1)	0.98
Familial Hypertension, N (%)	5 (11.6)	7 (18.9)	0.36
Previous myocardial infarction, N (%)	4(9.3)	4(10.8)	0.82
Previous coronary intervention, N (%)	13(30.2)	7(18.9)	0.24
Glomeruli filtration rate, mean±SD	54.47±9.08	50.46± 9.11	0.05
Serum Creatine, mean±SD (median)	1.19±0.26 (1.1)	1.25±0.25 (1.2)	0.23
Statin, N (%)	37(86.0)	33(89.2)	0.74
Beta blockers, N (%)	33(76.7)	27(73.0)	0.69
Calcium channel blockers, N (%)	15(34.9)	11(29.7)	0.62
ACE inhibitors or ARBs, N (%)	24(55.8)	18(48.6)	0.52
Aspirin, N (%)	41(95.3)	33(89.2)	0.40

*Values are given as number of patients (%) or mean± SD

**Table 2:** Type and number of vessels undergoing intervention in the omega-3 and control groups

**Target vessel**	**Control** **(n=43)**	**Omega 3** **(n=37)**	**p-value**
**LAD, N (%)**	16(37.2)	31(83.8)	<0.001
**LCX, N (%)**	15(34.9)	9(24.3)	0.30
**RCA, N (%)**	16(37.2)	10(27.2)	0.33
**OM, N (%)**	8(18.6)	4(10.8)	0.33
**Distal, N (%)**	4(9.3)	7(18.9)	0.21
**PDA, N (%)**	3(7.0)	1(2.7)	0.62*
**PLV, N (%)**	0(0.0)	1(2.7)	0.47*
**Number of vessel intervention, N (%)**			0.08
**One**	25(58.1)	17(45.9)		
**Two**	17(39.5)	14(37.8)	
**Three**	1(2.3)	6(16.2)	

LAD, Left anterior descending; LCX, Left circumflex; RCA, Right coronary artery; OM, Circumflex branch; PDA, Patent ductus arteriosus; PLV, Posterior left ventricular artery * Fisher exact test

**Table 3:** Hs-CRP levels (μg/mL) at baseline, and 24 h after PCI in the omega-3 and control groups

**hs-CRP**	**Control** **(n=43)**	**Omega 3** **(n=37)**	**p-value**
Baseline, median (IQR)	32.10 (11.60-41.90)	19.90 (7.60-38.15)	0.168
After intervention, median (IQR)	56.70 (26.07-79.10)	40.40 (13.15-74.95)	0.163
Baseline-24 h hs-CRP, median (IQR)	-22.10 [-40.60-(-0.19)]	-12.40 (-44.05-2.18)	0.432

hs-CRP, High sensitive C-reactive protein

**Table 4:** Comparison of 30-day MACE in the omega-3 and control groups

30 days MACE	Control (n=43)	Omega 3 (n=37)	p-value
Myocardial infarction/Angina, N (%)	6(14.0)	2(5.4)	0.27*
Revascularization, N (%)	4(9.3)	2(5.4)	0.68*
Total MACE, N (%)	10(27.9)	4(10.8)	0.05

MACE, Major Adverse Cardiac Event * Fisher exact test

Several experimental and clinical trials have shown the role of inflammation in the pathogenesis and progression of coronary artery disease.^9^ Studies suggest that CRP, a marker of systemic inflammation, reflects vascular wall inflammation with the predictive value for measuring adverse cardiac events after coronary interventions.^6^ The effect of preprocedural and postprocedural CRP levels in the evaluation of early and late clinical outcomes in conventional angioplasty and coronary artery stenting is well documented in the literature.^10,11^ Recent studies have particularity focused on the value of hs-CRP, a more sensitive measure of CRP, in the prediction of cardiac events in the setting of PCI. It has been shown that elevated hs-CRP level is associated with higher rates of cardiovascular adverse events such as stent restenosis after PCI. In a study on patients undergoing PCI for stable coronary disease, it was found that the inflammatory response expressed by hs-CRP levels was higher in patients undergone multi-vessel coronary intervention. Therefore, there seems to be a correlation between plasma hs-CRP levels and the extent of periprocedural artery injury.^12^

In another trial, plasma hs-CRP was found to be an independent predictor of non-target lesion progression in patients with stable angina after stent implantation; hence, the measurement of baseline hs-CRP may help to detect the patients who are at risk of rapid progression of coronary atherosclerosis.^13^

Patients with renal dysfunction are more likely to suffer from systemic atherosclerosis and thus cardiovascular events.^14^ In addition; higher rates of clinical and angiographic restenosis after coronary stenting have been reported in these patients. It has been revealed that impaired renal function is an independent risk factor of restenosis in CKD patients ^15^ and an incremental relationship between lower renal function and increased rate of MACE after PCI was detected. It was also suggested that CKD patients undergoing hemodialysis had a greater rate of repeat revascularization and mortality compared to the non-hemodialysis patients undergoing PCI.^16,17^

In many cases, DES has been reported to be more effective than bare-metal stents (BMS) due to the lower rate of restenosis and target vessel revascularization.^18^ Therefore; it has been suggested to perform coronary stenting using DES in CKD patients. Previous studies have investigated the potential effect of sirolimus-eluting stents on decreasing the need for repeating revascularization and also found no impact on lowering mortality in CKD patients after PCI.^19,20^ Furthermore, the efficacy of the paclitaxel-eluting stents needs to be studied in these patients. In a study on the relationship between the degree of renal function and long-term cardiovascular outcomes in patients using paclitaxel-eluting stent, it was demonstrated that the rates of cardiac death and revascularization were still significantly high in moderate CKD and dialysis patients even with the administration of DES. As a result, it was found that an interventional strategy with DES coupled with the traditional medical therapy statins, beta blockers, and angiotensin-II receptor blocking agents was still insufficient for moderate CKD and dialysis patients.^17^ Therefore, it is not supersizing that the optimal drug therapy with new agents might be able to improve prognosis of CKD patients treated with DES. Consequently, in this research, we evaluated the potential effect of omega-3, as a new strategy, in combination with standard regimen of PCI in this category of patients.

Over the past decade, there has been growing interest in studying the effects of anti-inflammatory agents in reducing the levels of hs-CRP and their effects on the clinical outcome of patients who had undergone PCI. The positive effect of statins on lowering hs-CRP, irrespective of their effects on other inflammatory markers or cholesterol, has been explored in various studies.^21,22^ Hence, several trials have reported the beneficial role of statins in decreasing hs-CRP and, therefore; enhanced clinical outcomes in patients undergoing PCI. One study showed the correlation between statin pretreatment and reduced myocardial necrosis at high-risk patients undergoing PCI.^23^ Likewise, in a large cohort study, it was found that statin therapy before PCI was associated with a significant mortality reduction after 30 days (early follow-up) and 6 months after the intervention (intermediate follow-up).^24^ The significant effect of pre-treatment with fluvastatin on reducing MACE was also reported in patients with average cholesterol levels undergoing their first successful PCI.^25^ Although non-significant, our study showed similar results like the above studies since both baseline hs-CRP and the difference between baseline and 24-h hs-CRP plus 30-day MACE were higher in control group compared to omega-3 group.

Regarding the positive cardiovascular effects of omega-3 as reported in several studies,^26-29^ in this trial, we evaluated the effect of omega-3 as an adjunctive therapy to the standard drug regimen before PCI in CKD patients. Omega-3 exerts its cardiac effects through several mechanisms including anti-platelet, improved responsiveness to anti-platelets such as aspirin and clopidogrel in patients with partial resistance as well as anti-lipidemic, and anti-inflammatory effect.^7^ As an anti-inflammatory agent, it has been shown to have beneficial effects on lowering different inflammatory biomarkers. The potential role of omega-3 in decreasing hs-CRP has been reported in several cases such as atherosclerotic^26^ and hemodialysis patients,^27^ irrespective of its anti-lipidemic effects. There are few reports on the positive effect of omega-3 in preventing peri-procedural myocardial damage^28,29^ and its effect on decreasing post-PCI hs-CRP in patients with normal kidney function,^8,26,27^ and also a few studies on assessing the effect of this supplement on the level of post-PCI cardiac necrosis markers in CKD patients.^30^ To the best of our knowledge, this study is novel in assessing the potential efficacy of omega-3 on the level of post-PCI hs-CRP and 30-day MACE in patients with moderate CKD (stage 3 based on K/DOQI stages). Our previous study on the effect of omega-3 on CRP in patients with normal kidney function, we demonstrated the positive effect of omega-3 in reducing post-PCI CRP at a dose of 1200 mg EPA and 400 mg DHA (two main fatty acids of fish oil).^8^Therefore, we used similar doses of EPA and DHA in this trial.

Our results also showed that although the downward trend in the elevation of 24-h hs-CRP in the omega-3 group was seen, there was no significant difference between the omega-3 and control groups. Several factors may contribute to this insignificance including low sample size or high LAD vessel involvement in omega-3 arm. To counter the confounding effect of more LAD distribution in the omega-3 group, we re-analyzed the data, separating those with or without LAD involvement. However, no significant variation was detected in differences between pre- and post-PCI hs-CRP in patients with LAD involvement among the two groups (*p* = 0.822). Also, it is possible that the standard therapy may lead to an insignificant effect of omega-3 on hs-CRP between the groups; however, standard PCI treatment could not be excluded from the regimen in the omega-3 group due to ethical considerations.

In addition, our results showed that premedication with a single dose of omega-3 could effectively decrease 30-day MACE in post-PCI patients with CKD. This effect may be explained by different mechanism other than anti-inflammatory for omega-3. For instance, omega-3 may still provide cardiac benefits through inhibition of platelet aggregation in CKD patients undergoing elective PCI (counting the important role of platelet aggregation in the pathogenesis of atherosclerosis), however; mechanistic studies are needed to prove this assumption.

With regards to the fact that there is a graded relationship between lower renal function and increased rate of MACE, our results disclosed that omega-3 still exerted its positive effect on decreasing 30-day MACE despite significantly lower eGFR in the omega-3 group compared to the controls.

### Study limitations

In this study, only a single dose of omega-3 was used. Treatment with higher doses and longer duration may have been be more effective. Moreover, we did not follow the patients for evaluating the long-term effect of omega-3 on MACE, as omega-3 may have long-term benefits. Small sample size is the other limitation of our trial.

## Conclusion

Our results revealed the positive effect of the omega-3 supplement on decreasing 30-day MACE; therefore, omega-3 may be considered as an effective adjunctive therapy to the standard drug regimen used before PCI. The evaluation of the effect of omega-3 on long-term MACE is recommended for future studies.

## Acknowledgments

This research, extracted from a thesis written by Bahram Movahed Nouri, was financially supported by Shiraz University of Medical Sciences (grant number: 93/1167). The authors would like to express their gratitude to Center for Development of Clinical Research of Nemazee Hospital for statistical analysis and the assistance of the staff of Kowsar and Alzahra Hospitals.

## Ethical Issues

The trial was approved by the ethics committee and registered by the Iranian Registry of Clinical Trials (IRCT) with an identifier code of IRCT2014122720441N1. All the participants signed an informed consent before enrolment in the study.

## Conflict of Interest

Authors declare no conflicts of interest.
